# Roles of Inflammation, Oxidative Stress, and Vascular Dysfunction in Hypertension

**DOI:** 10.1155/2014/406960

**Published:** 2014-07-20

**Authors:** Quynh N. Dinh, Grant R. Drummond, Christopher G. Sobey, Sophocles Chrissobolis

**Affiliations:** Vascular Biology & Immunopharmacology Group, Department of Pharmacology, Monash University, Wellington Road, Clayton, VIC 3800, Australia

## Abstract

Hypertension is a complex condition and is the most common cardiovascular risk factor, contributing to widespread morbidity and mortality. Approximately 90% of hypertension cases are classified as essential hypertension, where the precise cause is unknown. Hypertension is associated with inflammation; however, whether inflammation is a cause or effect of hypertension is not well understood. The purpose of this review is to describe evidence from human and animal studies that inflammation leads to the development of hypertension, as well as the evidence for involvement of oxidative stress and endothelial dysfunction—both thought to be key steps in the development of hypertension. Other potential proinflammatory conditions that contribute to hypertension—such as activation of the sympathetic nervous system, aging, and elevated aldosterone—are also discussed. Finally, we consider the potential benefit of anti-inflammatory drugs and statins for antihypertensive therapy. The evidence reviewed suggests that inflammation can lead to the development of hypertension and that oxidative stress and endothelial dysfunction are involved in the inflammatory cascade. Aging and aldosterone may also both be involved in inflammation and hypertension. Hence, in the absence of serious side effects, anti-inflammatory drugs could potentially be used to treat hypertension in the future.

## 1. Introduction

**Figure 1:**
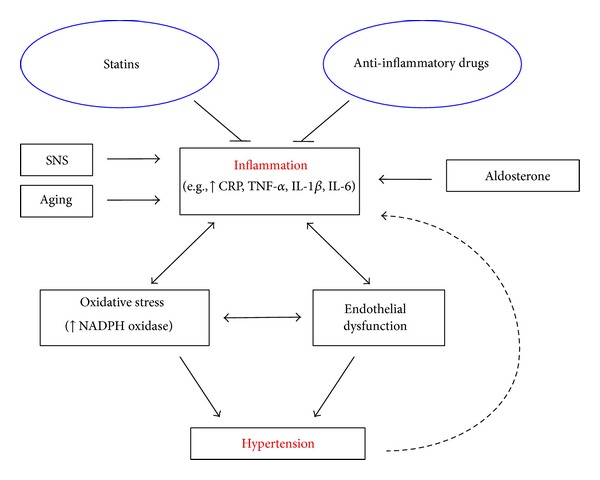
Schematic diagram illustrating the relationship between inflammation and hypertension and the contributing factors involved. Anti-inflammatory drugs and statins may be effective antihypertensive due to their anti-inflammatory properties.

Hypertension is the most common cardiovascular risk factor [1] and contributes to widespread morbidity and mortality worldwide [2]. Hypertension is a complex condition, and about 90% of cases are classified as essential hypertension, where the precise cause is unknown [3]. A small minority of hypertensive patients have secondary hypertension, in which a known factor is specifically responsible for raising blood pressure. Many secondary causes of hypertension include primary aldosteronism, obstructive sleep apnea, and renovascular disease [4]. An association between hypertension and inflammation has now been clearly demonstrated; however, it is presently unclear whether inflammation is predominately a cause or effect of hypertension. This brief review will describe evidence that inflammation might lead to the development of hypertension (Figure 1).

## 2. Inflammation in Human Hypertension

Current therapies for human hypertension include angiotensin II (Ang II) type 1 receptor (AT1R) inhibitors, angiotensin converting enzyme (ACE) inhibitors, diuretics, calcium channel antagonists, and *β*-blockers. Treatment with commonly used antihypertensives reduces the risk of total major cardiovascular events, and importantly, it appears that the larger the reduction in blood pressure, the larger the reduction in cardiovascular risk [5]. Insulin resistance contributes causally toward the pathogenesis of hypertension [6, 7]. Indeed, hypertension has been found to be associated with hyperinsulinemia and insulin resistance in humans [8]. Yet, while the above-mentioned therapies successfully lower blood pressure in most individuals, there are a group of patients who are resistant to such treatments. Furthermore, even when blood pressure targets are achieved, many hypertensive patients remain at risk for a cardiovascular event, which may be due to underlying inflammation.

Inflammation is a protective response to injury or infection. It is a complex process that involves inflammatory cells first identifying the affected tissue, leukocyte recruitment into tissue, elimination of the offending agent, and repair of the site of injury. Inflammation requires interactions between cell surfaces, extracellular matrix, and proinflammatory mediators [9]. Excessive inflammation can have detrimental effects and contribute to the progression of chronic and/or prolonged diseases such as atherosclerosis [10], rheumatoid arthritis [11], and systemic lupus erythematosus [12].

The acute phase protein, C-reactive protein (CRP), is involved in innate immune responses and has roles that include activating the complement system and enhancing phagocytosis [13]. CRP can stimulate monocytes to release proinflammatory cytokines such as interleukin-6 (IL-6), interleukin-1 beta (IL-1*β*), and tumour necrosis factor alpha (TNF-*α*) [14] and also endothelial cells to express intracellular adhesion molecule (ICAM)-1 and vascular cell adhesion molecule (VCAM)-1 [15], effects which will further promote inflammation.

CRP is considered the inflammatory marker with the strongest association with hypertension. It has been demonstrated in numerous clinical trials that hypertensive patients commonly have increased plasma CRP levels [16–21]. Both males and females participated in these studies and the ages of these patients varied from young [16] to middle aged [17]. Prehypertensive patients generally have higher plasma CRP levels than normotensive patients [22], and higher baseline CRP levels are reportedly associated with a higher risk of developing overt hypertension [23–25], consistent with the concept that systemic low-grade inflammation may precede hypertension. Systemic low-grade inflammation can be defined as a 2- to 3-fold increase in plasma levels of cytokines and acute phase proteins [26]. As discussed below, hypertensive patients have been reported to have higher plasma concentrations of proinflammatory cytokines. Nonhypertensive offspring of hypertensive parents tend to have higher serum CRP levels than offspring of nonhypertensive parents [27]. Studies have also demonstrated higher plasma IL-6 [28–30], IL-1*β* [31, 32], and TNF-*α* [28, 33, 34] levels in hypertensive patients compared to normotensive patients.

There is also evidence for involvement of immune cells in human hypertension. Patients with hypertensive nephrosclerosis have higher renal infiltration of CD4^+^ and CD8^+^ T cells than normotensive control patients [35]. Furthermore, C-X-C chemokine receptor type 3 (CXCR3) chemokines are well-known tissue-homing chemokines for T cells, and circulating levels of CXCR3 chemokines have been reported to be elevated in hypertensive patients [35]. Acquired immunodeficiency syndrome (AIDS) patients have reduced CD4^+^ T cells and the incidence of hypertension has been reported to be lower in AIDS patients than in HIV-negative participants. The highly active antiretroviral therapy (HAART) which can raise T cell levels increases the incidence of hypertension in AIDS patients similar to HIV-negative participants after treatment for less than 2 years, and the incidence of hypertension in AIDS patients is higher than in HIV-negative participants after treatment for 2 to 5 years [36]. In addition, it is becoming increasingly recognized that both neonatal and childhood health and disease are linked to the prenatal environment. Indeed, infants born following intrauterine inflammation are at increased risk of perinatal morbidity and mortality than infants born to healthy mothers [37–39].

## 3. Inflammation in Experimental Hypertension

Findings from animal studies have also suggested a role for inflammation in the pathophysiology of hypertension. Spontaneously hypertensive rats (SHR) are a genetic model of essential hypertension that develop hypertension as they age. SHR at 3 weeks of age are not yet hypertensive, yet their kidneys have higher levels of infiltrating lymphocytes and macrophages and activation of nuclear factor-kappaB (NF-*κ*B) than in Wistar Kyoto (WKY) normotensive control rats. These inflammatory changes in the kidneys continue to increase, together with blood pressure, with age in the SHR [40]. Junctional adhesion molecule (JAM)-1 is involved in leukocyte binding to the endothelium and has been found to be upregulated in the brainstem of SHR compared to WKY rats. Overexpression of JAM-1 in WKY rats resulted in elevated systolic blood pressure [41].

Macrophage colony-stimulating factor (m-CSF) acts as a chemotactic factor for monocytes and regulates both effector functions of mature monocytes and macrophages, and production of cytokines. Mice deficient in m-CSF [42], IL-6 [43], TNF-*α* [44], or interleukin-17 (IL-17) [45] develop a lower blood pressure in response to a hypertensive dose of Ang II compared with control mice. RNA interference knockdown of IL-6 in rats has also been shown to inhibit hypertension [46]. Suppression of NF-*κ*B reportedly inhibits the increase in blood pressure that normally occurs in SHR [47]. Lipopolysaccharide (LPS)- endotoxin from gram-negative bacteria elicits a strong immune response and intraperitoneal injection of LPS is a well-characterised model of systemic inflammation in rodents. In rats, LPS-induced increases in plasma levels of CRP, TNF-*α*, and IL-1*β* are associated with an increase in blood pressure [48]. Inhibition of Cox-2 in LPS-treated rats inhibited the increase in blood pressure, suggesting that inflammation in response to LPS treatment contributed to the hypertensive effect [48]. Offspring with prenatal exposure to LPS [49, 50] or IL-6 [51] have higher blood pressure than control offspring.

Immune cells have been implicated to play a role in the development of hypertension. RAG-1 deficient mice, which lack T and B cells, do not develop hypertension in response to Ang II [52], DOCA-salt [52], or chronic stress [53]. Adoptive transfer of T cells but not B cells restored the hypertensive effect in RAG-deficient mice [52]. An analogous effect has been reported to occur in macrophage-depleted mice in response to Ang II infusion [54].

T cells have been demonstrated to express AT1R (the main target receptor of Ang II), ACE, angiotensinogen, renin, and the renin receptor, all important components of the renin angiotensin aldosterone system (RAAS) [55]. The RAAS is a key regulator of blood pressure, and excessive stimulation of this system can cause hypertension. While T cells are capable of producing Ang II [56], which can cause vasoconstriction, stimulate inflammation, and aldosterone production [57], the role of T cell-derived Ang II in such effects is uncertain. There is some evidence that inflammation can overstimulate the RAAS. Inflammatory markers such as CRP [58], TNF-*α*, and IL-1*β* [59] have been found to upregulate AT1R. Furthermore, central administration of LPS to rats increases AT1R mRNA expression in the hypothalamus [60].

Given the importance of this recent concept that immune cells are involved in hypertension, we will briefly discuss a hypothesis presented in the literature by Harrison's group regarding how immune cells are activated in hypertension. Hypertensive stimuli, including salt, overactivity of the RAAS, oxidative stress, and inflammation lead to an initial elevation in blood pressure (mainly because of central actions but also due to endogenous hormones such as Ang II and aldosterone), which results in protein modifications. These altered proteins are no longer recognized as self (i.e., they serve as neoantigens), and T cells are activated. T cell-derived signals promote entry of macrophages (and other inflammatory cells) into the vasculature and kidney which results in cytokine release. In the vasculature, activated T cells promote vasoconstriction and remodeling. Together with the promotion of sodium and water retention in the kidney, more severe hypertension can result [61, 62].

## 4. Inflammation and Endothelial Dysfunction in Hypertension

**Figure 2:**
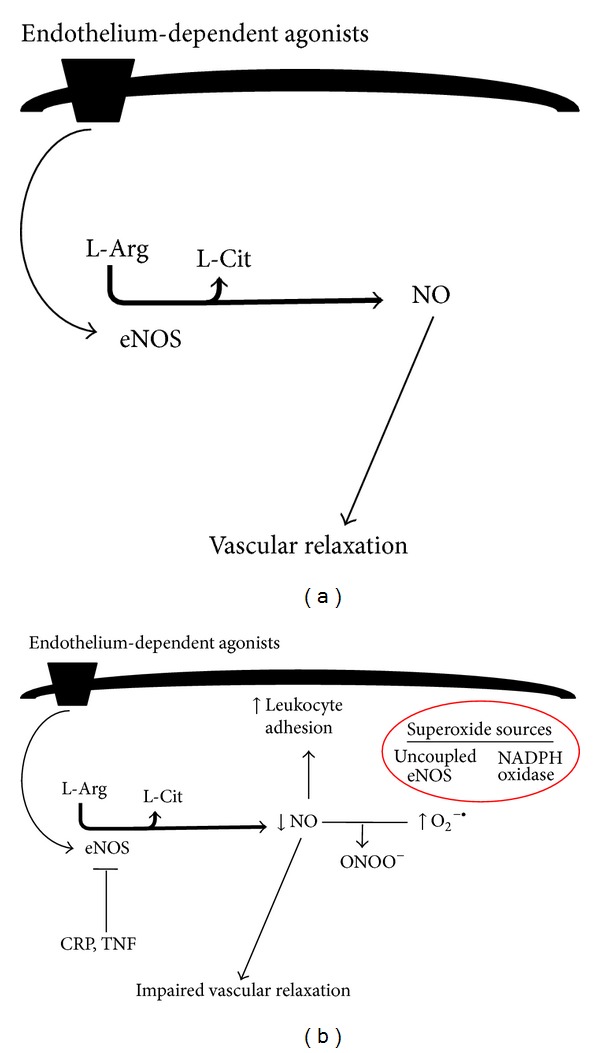
(a) Diagram illustrating endothelium-dependent relaxation during health. (b) Diagram illustrating mechanism by which inflammation and oxidative stress cause endothelial dysfunction. Inflammatory mediators such as CRP and TNF destabilise eNOS mRNA (thus inhibiting eNOS). NO protects endothelium by inhibiting leukocyte adhesion; thus, impaired NO function results in increased leukocyte adhesion. Increased superoxide (derived from NADPH oxidase or uncoupled eNOS) impairs NO bioavailability and leads to impaired vascular relaxation. The figure is based on text.

One potential mechanism by which inflammation may promote hypertension is by causing endothelial dysfunction. The endothelium is a single cell layer that lines the luminal surface of blood vessels and is involved in regulation of vascular tone and structure. Nitric oxide (NO) derived from endothelial nitric oxide synthase (eNOS) is a signalling molecule important in regulating vascular tone. When NO is released from endothelial cells it causes smooth muscle relaxation and subsequent vasodilation (Figure 2) [63]. Endothelial dysfunction may contribute to increased systemic vascular resistance and thus lead to the development of hypertension and is commonly manifested as impaired endothelium-dependent vasodilation due to an imbalance between vasoconstrictors and vasodilators [64]. Inflammation can alter the rates of synthesis and degradation of vasoconstrictors and vasodilators including NO, and impaired NO bioactivity is associated with hypertension. Rats chronically treated with the NO synthase inhibitor, N-nitro-L-arginine methyl ester, have higher blood pressure than controls [65]. Inflammation has been shown to downregulate NO synthase activity. For example, CRP [66] and TNF [67] have both been demonstrated to attenuate NO production by destabilising eNOS mRNA, which reduces NOS protein expression, and inhibition of TNF restores endothelial-dependent vasodilation in humans [68] (Figure 2). IL-17 has been reported to cause endothelial dysfunction by activating Rho-kinase, which leads to phosphorylation of the inhibitory eNOS residue, threonine 495 [69]. Inhibition of eNOS increases vascular tone [70] and consistent with this, Rho-kinase has been shown to contribute to cerebral vascular tone* in vivo* and this is enhanced during chronic hypertension. In contrast, protein kinase C was not found to contribute to cerebral vascular tone in either normotensive or hypertensive animals [71]. Increased serum CRP levels also correlate with reduced NO bioavailability in coronary artery disease patients [72]. Mice deficient in m-CSF were found to develop less endothelial dysfunction and vascular oxidative stress in response to Ang II compared to wild type mice [42].

Importantly, normal healthy endothelium exerts anti-inflammatory effects such as NO-dependent inhibition of leukocyte adhesion [73]. Inhibition of eNOS activity augments expression of leukocyte adhesion molecules and chemokines such as monocyte chemotactic protein 1 (MCP-1) [74, 75]. Furthermore, gene therapy to augment vascular NO synthase activity attenuates hypercholesterolemia-induced leukocyte adhesion molecule expression and monocyte infiltration [76]. Therefore, endothelial dysfunction can further exacerbate vascular inflammation, which may in turn contribute to the development of hypertension.

## 5. Inflammation and Oxidative Stress in Hypertension

Chronic inflammation can also trigger oxidative stress, which has been associated with hypertension [77]. As mentioned, inflammation is the primary immune response to eliminate pathogens or to repair tissue damage. Innate immune cells, such as neutrophils and macrophages, produce reactive oxygen species (ROS) such as superoxide and hydrogen peroxide in order to kill pathogens [77]. Nicotinamide adenine dinucleotide phosphate-oxidase (NADPH) oxidase is a major source of ROS in immune cells and also in the vasculature [78]. Inflammatory processes continue until the pathogens are destroyed or the tissue repair process has been completed. However, sustained inflammation can lead to an overproduction of ROS. Oxidative stress (defined as an imbalance between the production and breakdown of ROS) is a major cause of endothelial dysfunction, primarily through reducing NO bioavailability via the direct chemical reaction of superoxide with NO, resulting in the formation of peroxynitrite [79]. The reaction between superoxide and NO is faster [80] than the breakdown of superoxide via superoxide dismutase [81]. Furthermore, peroxynitrite formation may result in further impairment of NO levels and enhanced oxidative stress by inhibiting eNOS activity through oxidation of 4-tetrahydrobiopterin (BH_4_), a cofactor of eNOS. This leads to eNOS uncoupling, where eNOS produces superoxide instead of NO [82]. Excessive ROS levels can also induce cellular damage by interacting with DNA, lipids, and proteins [83], which may further impair vascular structure and function. Immune cells such as T cells, macrophages, and neutrophils express NADPH oxidase subunits and produce ROS. In the setting of Ang II-induced hypertension, T cells express higher levels of p47^phox^, p22^phox^, and NOX2, components of NOX2 oxidase. Furthermore, adoptive transfer of T cells deficient in NADPH oxidase results in lower superoxide production and hypertension in response to Ang II [52]. Oxidative stress can promote inflammatory processes by activating transcription factors such as NF-*κ*B [84]. CRP levels have been shown to correlate with the level of oxidative stress in inflammatory cells from hypertensive patients [85].

The kidney is an important organ involved in regulating blood pressure, and chronic kidney disease is one of the most common causes of secondary hypertension [86]. Elevated renal oxidative stress can be seen in the early stages of chronic kidney disease [87], and inflammation [88] and oxidative stress [87] increase as renal dysfunction progresses. Prehypertensive SHR from 2-3 weeks of age have elevated renal inflammation and oxidative stress compared to age-matched WKY rats [89]. Renal artery stenosis results in reduced renal perfusion and pressure at the afferent arteriole, thus stimulating the release of renin and, hence, activation of the RAAS [90]. Renal artery stenosis can cause renovascular hypertension, a secondary form of hypertension due to kidney dysfunction. Indeed, reduced blood flow to the kidney decreases renal function and can lead to chronic kidney disease [91]. Renal artery stenosis is the most common primary disease of renal arteries, and up to 90% of cases are caused by atherosclerosis [92], a chronic inflammatory disease that predominately occurs in large arteries, and oxidative stress plays a major role in its development [93].

Overall, with oxidative stress being able to act as a key trigger of both inflammation and hypertension, it remains unclear whether inflammation is predominately a cause or effect of hypertension, with evidence to support either scenario in a likely vicious cycle.

## 6. The Sympathetic Nervous System and Inflammation in Hypertension

Sympathetic nervous system (SNS) activation is a common feature of hypertension and can contribute to the development of hypertension [94]. Essential hypertension patients are reported to have increased renal sympathetic outflow [95]. Autonomic dysfunction is characterised by increased sympathetic and decreased parasympathetic activity, and the SHR has been shown to be a good rodent model of human autonomic dysfunction [96]. The SNS innervates primary and secondary lymphoid organs and most immune cells express receptors for catecholamines such as noradrenaline [97]. The SNS can enhance inflammatory responses. For example, deletion of extracellular superoxide dismutase in the circumventricular organs of mice increased sympathetic outflow, modestly elevated blood pressure and increased T cell activation [98]. Renal sympathetic nerves have been suggested to play a role in kidney inflammation. Rats that have undergone renal sympathetic denervation have reduced renal macrophage levels and cortical TNF expression [99]. Neuroinflammation has been associated with increased sympathetic drive during cardiovascular disease. Inflammation may also promote SNS activation. Central administration of LPS to rats is reported to increase renal sympathetic drive and blood pressure [60]. Blood-borne pro-inflammatory cytokines may also act on receptors in the microvasculature of the brain to induce Cox-2 activity and the production of prostaglandins which penetrate the blood brain barrier to activate the SNS [100]. Overall, these studies suggest a potentially important link between the SNS and inflammation in the development of hypertension.

Increased sympathetic drive to the kidneys causes the release of renin and subsequently raises blood pressure [101]. Catheter-based renal denervation is a promising therapeutic approach to treat resistant hypertension [102]. Catheter-based renal denervation involves the application of radiofrequency energy in short bursts along the main renal arteries to disrupt the renal nerves. However, the recent SYMPLICITY HTN-3 clinical trial has reported that renal denervation does not result in a significant reduction in systolic blood pressure in resistant hypertensive patients with systolic blood pressure readings above 160 mmHg when compared to sham control [103]. The previous SYMPLICITY HTN clinical trials [102, 104] had reported that renal denervation can cause large reductions in blood pressure; however, these clinical trials were unblinded, had small sample sizes, and lacked sham controls as opposed to SYMPLICITY HTN-3. This does not necessarily mean that renal denervation is ineffective in lowering blood pressure as SYMPLICITY HTN-3 used the Medtronic catheter and there are other catheters available; hence, future clinical trials could study the use of other catheters to lower blood pressure. Currently there is a clinical trial examining Covidien's OneShot device [105].

## 7. Aging and Chronic Inflammation

More than half of the elderly (above 65 years of age) have hypertension [106], and the prevalence of hypertension increases with age [107]. Secondary causes of hypertension such as obstructive sleep apnea, chronic kidney disease, and renal artery stenosis, which are all associated with inflammation, are highly prevalent in the elderly [108]. Chronic low-grade inflammation commonly occurs with aging and this has been termed “inflammaging” [109]. Inflammaging is characterised by an imbalance of proinflammatory markers and anti-inflammatory markers. Levels of proinflammatory markers such as IL-6, TNF-*α*, and CRP are elevated, while anti-inflammatory cytokines such as interleukin-10 are reduced [110]. Inflammaging is believed to be caused by continuous lifelong exposure to antigens, due either to infection and/or nonpathogenic antigens.

## 8. Aldosterone and Inflammation

Patients with primary aldosteronism have elevated aldosterone levels, and as mentioned earlier, primary aldosteronism is a common secondary cause of hypertension. More than 10% of hypertensive patients have raised aldosterone levels [111], and drugs that block the mineralocorticoid receptor (MR), the main target receptor of aldosterone, are used to treat hypertension that is resistant to ACE inhibition and AT1R antagonism [112]. Aldosterone is involved in the RAAS whereby a fall in blood pressure under physiological conditions leads to Ang II generation which, through its action on the AT1R in the adrenal zona glomerulosa, stimulates the release of the mineralocorticoid, aldosterone. Aldosterone activates the MR in the distal renal tubule of the kidney to increase sodium and water retention, and potassium excretion, leading to an increase in blood volume and thus blood pressure [113]. Actions of aldosterone were, until recently, believed to be restricted to the kidney, but it is now understood that aldosterone can target other tissues relevant to blood pressure control, including the brain [114], vasculature [115], and heart [116].

Aldosterone has been reported to exert proinflammatory effects that appear to be MR-mediated. Administration of exogenous aldosterone to experimental animals results in elevated levels of ICAM-1, MCP-1, and TNF-*α* in coronary arteries [117], and increased vascular infiltration of macrophages and lymphocytes [118]. In the heart, aldosterone can increase vascular expression of ICAM-1, MCP-1, osteopontin, and COX-2, which can be blocked by the MR antagonist, eplerenone [116], indicating involvement of the MR. Proinflammatory effects of aldosterone have also been reported to occur in the kidney, where aldosterone and salt treatment caused MR-dependent leukocyte infiltration and elevation of osteopontin, IL-6, IL-1*β*, and MCP-1 levels [119]. Association between aldosterone and inflammation has been reported in essential hypertensive patients, where high plasma aldosterone levels were correlated with high levels of circulating CRP and leukocytes [120].

## 9. Anti-Inflammatory Drugs and Hypertension

Currently, anti-inflammatory drugs are not used to treat hypertension. Of the different classes of anti-inflammatory agents, immunosuppressant drugs could potentially be used to treat hypertension. Mycophenolate mofetil, which blocks T cell and B cell proliferation by inhibiting inosine monophosphate dehydrogenase, has been demonstrated to reduce hypertension in SHR [121], in Dahl salt-sensitive rats (another rodent model of hypertension) [122, 123], and in psoriasis and rheumatoid arthritis patients [124]. Another immunosuppressant, tacrolimus, a calcineurin inhibitor which blocks T cell activation, is reported to reduce hypertension in Dahl salt-sensitive rats [125]. Furthermore, chronic kidney disease patients with hypertension and who are on immunosuppressant drugs were found to require less antihypertensive medication than patients who were not taking immunosuppressant drugs [126]. Based on these studies, T cells may be a potential target in treating hypertension. T cells are an important component of the immune system and are involved in various aspects such as regulating immune responses by secreting anti- and proinflammatory cytokines. Ang II and aldosterone have proinflammatory effects; hence, targeting the RAAS could also target inflammation in hypertension. Ang II has been demonstrated to stimulate T cell proliferation and T cells deficient in the AT1R proliferate much less than T cells from wild type mice [127]. Vaccines that target the RAAS are currently in development to treat hypertension. The CYT006-AngGb vaccine which targets Ang II has been reported to reduce blood pressure in patients with mild to moderate hypertension with no serious safety issues in a phase IIa clinical trial [128]. Recently, the ATRQ*β*-001 vaccine which targets the AT1R has been found to be successful in lowering blood pressure in Ang II-induced hypertensive mice and SHR [129]. However, further studies are required to demonstrate the long-term safety of vaccines which target the RAAS, as one of the earlier vaccines developed which targeted renin was found to cause fatal autoimmunity [130].

Immunosuppressants can have serious side effects [131]; hence, their potential use to treat hypertension should be carefully considered and is not currently justified. Moreover, it is noteworthy that nonsteroidal anti-inflammatory drugs can increase blood pressure by causing sodium retention [132]. The use of immunosuppressants to treat treatment-resistant hypertension could possibly be justified. Treatment-resistant hypertension is defined as blood pressure that is above the patient's goal, despite treatment with at least three different classes of antihypertensive drugs, including a diuretic, at optimal doses. Patients with controlled blood pressure, and taking at least four antihypertensive medications, are also considered to be treatment-resistant [133]. It is difficult to determine the exact prevalence of treatment-resistant hypertension, but clinical trials have suggested that this may include up to 30% of hypertensive patients [134]. Currently, there are few therapeutic options available to treat resistant hypertension.

Statins were developed to inhibit cholesterol synthesis by blocking HMG-CoA reductase, but these drugs may also have anti-inflammatory effects and can cause a small reduction in systolic blood pressure in hypercholesterolemic patients, an effect that is greater in patients with a higher baseline blood pressure [135]. Statins can reduce levels of proinflammatory cytokines such as IL-1, IL-6, TNF- *α*, ICAM-1, and CRP [136]. Even in participants without hyperlipidemia, rosuvastatin has been shown to reduce CRP levels in association with a lower incidence of cardiovascular events [137]. The anti-inflammatory effects of statins may occur through the mevalonate pathway (i.e., HMG-CoA reductase pathway), which is responsible for cholesterol synthesis [138]. Pitavastatin has been shown to reduce IL-6 and IL-8 secretions and mRNA expression of IL-6, IL-8, and granulocyte macrophage colony-stimulating factor (GM-CSF) in LPS-stimulated human bronchial epithelium cells. These effects were inhibited by treatment with mevalonate, the initial product of HMG-CoA reductase [139]. The anti-inflammatory effects of statins may also occur through NO. Statins have been shown to enhance mRNA and protein expression of eNOS in human endothelial cells by inhibiting Rho-kinase geranylgeranyl-phosphorylation [140], and as discussed earlier NO has protective anti-inflammatory effects. Statins may also improve eNOS coupling by reducing plasma asymmetrical dimethylarginine (ADMA) [141], as ADMA has been associated with eNOS uncoupling [142].

## 10. Conclusion

It is unclear whether inflammation is a cause or effect of hypertension, but as discussed in this review there is evidence from human and animal studies suggesting that inflammation can lead to the development of hypertension. Oxidative stress and endothelial dysfunction are known to be associated with inflammation and can contribute to hypertension, at least in part, by exacerbating the inflammatory response. Other factors that contribute to hypertension such as SNS activation, aging, or aldosterone are also associated with inflammation (summarized in Figure 1). Future studies should focus on whether anti-inflammatory drugs are beneficial in reversing hypertension, oxidative stress, and endothelial dysfunction in experimental models of hypertension. Hence, in the absence of serious side effects, anti-inflammatory drugs could potentially be used not only to treat hypertension in the future but also to treat other cardiovascular diseases by minimizing the impact of oxidative stress and endothelial dysfunction.
